# A study of the association of cognitive abilities and emotional function with allergic disorders in young women

**DOI:** 10.1186/s12905-021-01345-x

**Published:** 2021-05-17

**Authors:** Mohammad Fereidouni, Hadis Rezapour, Mansoore Saharkhiz, Sara Mahmoudzadeh, Malaksima Ayadilord, Masoumeh Askari, Samira Karbasi, Arefeh Abbaszadeh, Zahra Sadat Hoseini, Gordon A. Ferns, Afsane Bahrami

**Affiliations:** 1 Cellular and Molecular Research Center, Birjand University of Medical Sciences, Birjand, Iran; 2 Student Research Committee, Birjand University of Medical Sciences, Birjand, Iran; 3 Department of Immunology, Faculty of Medicine, Birjand University of Medical Sciences, Birjand, Iran; 4 Department of Anatomical Sciences, Faculty of Medicine, Birjand University of Medical Sciences, Birjand, Iran; 5 Cardiovascular Diseases Research Center, School of Medicine, Birjand University of Medical Sciences, Birjand, Iran; 6 Department of Psychology, University of Birjand, Birjand, Iran; 7 Division of Medical Education, Brighton and Sussex Medical School, Falmer, Brighton, BN1 9PH Sussex UK

**Keywords:** Anxiety, Asthma, Depression, Insomnia, Quality of life

## Abstract

**Background:**

Allergic disorders may have a bidirectional causal relationship with mental disorders. In this cross-sectional study, we aimed to assess the associations between cognitive abilities and emotional function tests and quality of life with the presence of allergic disease in young women.

**Methods:**

A diagnosis of allergic disorders, comprising allergic rhinitis (AR), asthma and atopic dermatitis (AD), was confirmed by a specialist in allergy. The presence and severity of depression, anxiety, stress, insomnia and sleepiness were evaluated using validated questionnaires. Cognitive abilities and quality of life were assessed using standard instruments.

**Results:**

Among 181 female young participants, the prevalence of AR, asthma and AD were 26.5%, 2.8%, and 14.9% respectively. The AR group had higher scores than the non-AR group for depression, anxiety, insomnia, and lower scores for physical and mental health-related quality of life. Moreover, the AD cases had higher scores on the depression and stress scale compared to those without it (*p* < 0.05). Asthmatic patients also had significantly higher insomnia severity and lower physical health-related quality of life than non-asthmatic.

**Conclusion:**

There was a high prevalence of psychological/psychiatric disorders that included: anxiety, and sleep problems among allergic women, and a reduced quality of life that may be associated with it.

## Background

Atopy is a predisposition to mount an excessive immunological response to a wide range of allergens, causing CD4 + Th2 differentiation and prolonged synthesis of immunoglobulin E (IgE). Atopy may arise through aberrant regulation by T helper cells and suppressor T lymphocytes that normally assist in the generation of IgE by plasma cells. The clinical effect of this is the propensity to develop hypersensitivity reactions to specific epitopes. Genetics and environmental factors contributed to the development of atopy [1].

There has been a steady rise in the prevalence of atopy in some countries, in particular allergic asthma, allergic rhinitis (AR), and atopic dermatitis (AD), in recent decades. Allergic disease is common globally, with different manifestations affecting more than half of the general population [2, 3]. Allergic rhinoconjunctivitis is a chronic IgE-induced inflammation in the nasopharynx with typical manifestations include nasal congestion, rhinorrhea, sneezing, as well as itching [4].

Asthma is a chronic inflammatory disorders of the airways described as a reversible airflow obstruction and bronchial hyper-sensitivity affecting 300 million people worldwide [5, 6]. Eczema is a pruritic chronic skin disorders related to defective skin barrier extended immunological responses to environmental stimuli [7, 8].

It is possible that allergic disorders have a bidirectional causal relationship with mental disorders [9]. For instance, psychiatric disorders are common among asthmatics and are closely related to poor asthma control and quality of life [10]. Atopy-related conditions may cause psychological distress, which, in turn, may strengthen the manifestations of atopy. However, the reported relationship between atopy and psychological distress has been inconsistent. Mood and anxiety disorders, or major depressive disorder have been reported to be related to a higher risk of asthma [11–13]. A recent systematic review of 51 studies showed a significant relationship between AR and depression [14]. However, other reports found weak, or no associations, between anxiety or depression and atopy [15]. For instance, population-based studies in Australia and Asia demonstrated controversial findings regarding the association between AR and depression [16, 17]. In another study, anxiety was found to be associated with asthma and AR, but not with AD, and depression was not associated with any of these three atopic conditions [18].

Insomnia is defined as having difficulty falling asleep, maintaining sleep or having poor sleep quality despite a sufficient opportunity for sleep and together with daytime symptoms i.e. fatigue, irritability, and lower concentration. Sleep quality has been reported to differ between those with and without atopy [19, 20]. Health-related quality of life is a crucial patient outcome which evaluates how a patient’s health influences physical or mental well-being. Several psychosocial and emotional factors may be related with poor control of atopic symptoms and may affect the quality of life, but they have received little attention.

Psychiatric comorbidities may, however, exacerbate or inhibit symptom perception in chronic atopy. They may affect the pathophysiology of atopic disorders via downstream effects on the central nervous system (CNS), via the hypothalamic–pituitary–adrenal (HPA) axis that causes a cytokine response, that leads to a chronic pro-inflammatory state [21].

Cognitive impairment, depression, anxiety, stress, and sleep problems are common and important psychological disorders, but studies that have evaluated the association between these complications in the allergic cases have been inconsistent particularly in women. Female sex has previously been reported to be related with worse allergy control and worse allergy-related quality of life as well as increased rates of health care visits, and medication use [22, 23]. Hence, the purpose of the present study was to evaluate associations between cognitive abilities and emotional function tests and quality of life with the presence of allergic disorders among young women in East of Iran.

## Methods

### Study design

This cross-sectional study was performed in a group of female students in the city of Birjand, in eastern Iran, between December 2019–January 2020 [24]. Participants were chosen from 5 different universities in Birjand, using a multistage cluster sampling method. The sample size was calculated to achieve 80% power and *α*′ = 0.05 using data from a previous investigation [25]. To limit heterogeneity and control for potential confounding factors, only unmarried, apparently healthy women, aged between 18 and 25 years were recruited. The exclusion criteria included: the presence of any acute or chronic systemic disorders and taking any medication. The Ethics Committee of Birjand University of Medical Sciences approved the study (Code: 4891). All study participants gave written informed consent.

### Phenotypic characterization of allergic conditions

Diagnosis of allergic disease was performed by same specialist in allergy, by assessment of clinical history, physical examination, skin-prick testing (SPT) and laboratory assessment in allergy and immunology clinic. SPT for common allergens was conducted by an expert immunologist with standard protocols as described previously [26]. Non-allergic groups were participants having neither allergic sign/manifestations nor any sensitivity to allergens in the SPT. Total serum IgE levels were quantified by using an Elisa kit (Euroimmun, Germany) in accordance with the manufacturer’s protocols.

### Evaluation of cognitive abilities

Cognitive performance was assessed using the Cognitive Abilities Questionnaire (CAQ). This questionnaire includes 30 items which are rated from 1 to 5 to yield a total score ranging from 30 to 150. Higher scores imply better cognition performance. The CAQ evaluates seven abilities task including memory (6 items), inhibitory control and selective attention (6 items), decision making (5 items), planning (3 items), sustained attention (3 items), social cognition (3 items) and cognitive flexibility (4 items) [27].

### Assessment of depression, anxiety and stress

Depression anxiety and stress scale (DASS-21) is a most commonly used, valid and reliable instrument for evaluating negative emotions. This questionnaire comprises 21-items with 3 sub-scales in which each items is rated on a 4-point Likert scale (0–3) measuring depression, anxiety and stress. Because the DASS-21 is the brief version of DASS-42; the total score of each subscale must be doubled [28, 29]. Higher score related with severe negative emotions. The reliability and validity of DASS-21 for Iranian population was approved previously [29, 30]. Depression, anxiety, and stress scores were divided into two categories (No/Minimal state and some degree of disorder) according to the scores obtained for each subscale as follows: No (≤ 9) or some degree of depression (> 9); No (≤ 7) or some degree of anxiety (> 7); and No (≤ 14) or some degree of stress (> 14).

### Quality of life

The Short Form-12 Health Survey (SF-12) derived from the SF-36 is one of the most popular generic instrument for measurement of physical and mental components of quality of life [31]. The SF-12 consists of twelve questions covering 8 domains of personal health which higher scores represented better health related quality of life. The Persian version of SF-12 which has good reliability and validity for Iranian population was used in this study [32]. A score below the median cut off for the QoL score was considered to be a low QoL.

### Assessment of sleep pattern

#### Insomnia severity

The ISI was first developed by Morin et al. and has been used extensively in the last decades [33]. The ISI is a seven-item self-reported questionnaire measuring the nature, severity of insomnia and associated sleep problems such as sleep dissatisfaction. A 5-point Likert scale is used to score each question (0 = No, to 4 = very severe), providing a sum score between 0–28. Based on acquiring scores, individuals were categorized as: no clinically significant insomnia (0–7), or mild to severe degree of insomnia (8–28). The Persian version of the ISI has good reliability and validity for Iranian population was used in current study [34].

#### Sleepiness severity

The Epworth sleepiness scale (ESS) is an 8-item self-reported questionnaire to evaluate the average degree of daytime sleepiness propensity. Each item rated on 4-point Likert scale (0–3) to measures the habitual likelihood to fall asleep in eight daily life conditions [35]. Higher scores of the ESS reflected a greater average tendency to fall asleep. Normal values range from 0 to 10, score of 10–16 indicated mild to moderate sleepiness and more than 16 defined to severe sleep apnea or narcolepsy [36].

### Assessment of covariates

Demographic and anthropometric variables including: age, height, weight, waist and hip circumference were gathered by an expert nurse. Then, body mass index (BMI) was calculated as weight (kg) divided to height ^2^(m). Waist-to-hip ratio (WHR) was obtained by dividing waist circumference by hip circumference. Dietary intake of participants was gathered by a valid and reliable semi quantitative food frequency questionnaire (FFQ) consisting 65 food items in order to estimation of dietary intakes of subjects [37, 38] and then energy intake for each of them was estimated.

### Statistics

All of variables were normally distributed by using Kolmogorov–Smirnov test (*p* value for cognitive abilities, depression, anxiety, stress, quality of life, insomnia and sleepiness was 0.09, 0.051, 0.067, 0.21, 0.18, 0.052, 0.61) and were analyzed by applying parametric statistical tests. Parametric and non-parametric continuous variables as well as categorical indices demonstrated by Mean ± Standard deviation (SD), median and interquartile range (non-parametric variables), and number (percent), respectively. Independent sample T-test or Mann–Whitney test was used for comparison of continuous variables between 2 groups. Regression analysis was used to evaluate the predictive value of having allergy for emotional complications (as the dependent variables). In the analysis, anxiety and insomnia were divided into two categories (no/minimal status or some degree of disorder) regarding the scores and individuals in the first group (no/minimal status) were set a reference group. Then, binary logistic regression analyses were recruited to estimate the odds, as showed by the odds ratio (OR). A *p* < 0.05 was considered to be statistically significant. All of analysis performed by using SPSS for Windows version 16.0.

## Results

One hundred and eighty-one female students, between 18 and 27 years of age (mean: 20.7 ± 2.2 y) were recruited to this study. Among the 181 participants, fifty four (29.8%) individuals had at least one type of allergic disease. In total, AR, asthma and AD were present in 48(26.5%), 5(2.8%) and 27 individuals (14.9%), respectively. One hundred and twenty-seven (70.2%) individuals did not have any allergic disorders and were considered as non-allergic cases. Demographic characteristics of study participants were shown in Table 1. Allergic and non-allergy groups had similar mean age, BMI and WHR (*p* > 0.05). But, serum IgE levels significantly increased in atopy cases (*p* = 0.001).

**Table 1:** Demographic characteristics of study participants

Variables	Any allergy (Allergic Rhino-conjunctivitis, Eczema, Asthma)		
	Yes 54(29.8%)	No 127(70.2%)	*p* value
Age (year)	20.6 ± 1.4	21.1 ± 2.0	0.06
BMI (kg/m^2^)	20.8 ± 3.0	20.8 ± 2.8	0.98
WHR	0.74 ± 0.04	0.73 ± 0.04	0.82
IgE (U/mL)	151.6(53.7–402)	37.9(11.5–253)	**0.001**

Data presented as Mean ± SD (parametric variables) or median and interquartile range (non-parametric variables). By using independent sample *t*-test or Mann–Whitney test

The significance of bold values is *P* < 0.05

*BMI* body mass index, *WHR* waist-to-hip ratio, *IgE* immunoglobulin E

Allergic cases had higher anxiety and insomnia scores, and lower mental health related quality of life scores compared to those without any allergy (*p* < 0.05; Table 2). No significant difference was found between atopy and non-atopy groups in terms of cognitive abilities tasks (*p* > 0.05).

**Table 2:** Association between neuropsychological tests with different allergic disorders

Variables	Any allergy (Allergic Rhino-conjunctivitis, Eczema, Asthma)		
	Yes 54(29.8%)	No 127(70.2%)	*p* value
Memory	25.3 ± 3.4	25.7 ± 3.5	0.39
Inhibitory control and selective attention	21.7 ± 4.1	22.2 ± 3.8	0.38
Decision making	18.7 ± 3.4	19.1 ± 4.0	0.43
Planning	10.7 ± 2.9	11.5 ± 2.7	0.07
Sustain attention	9.4 ± 2.4	9.6 ± 2.4	0.69
Social cognition	10.6 ± 2.0	10.7 ± 2.2	0.38
Cognitive flexibility	14.6 ± 2.7	14.4 ± 2.9	0.63
Total cognitive ability task	110.9 ± 13.6	113.3 ± 14.9	0.27
*Dass-21*			
Depression	12.1 ± 9.4	9.5 ± 7.9	0.06
Anxiety	9.9 ± 6.7	7.4 ± 5.3	**0.009**
Stress	17.8 ± 10.0	16.8 ± 9.9	0.52
*Quality of life*			
Physical health	15.5 ± 2.6	16.2 ± 2.4	0.06
Mental health	15.9 ± 3.5	17.1 ± 3.9	0.036
SF-12 score	31.5 ± 5.0	33.4 ± 5.0	0.018
*Sleep pattern*			
Insomnia score (ISI)	7.3 ± 6.9	4.5 ± 6.3	**0.006**
Daytime sleepiness score (ESS)	6.7 ± 6.0	5.8 ± 5.7	0.31

Data presented as Mean ± SD. By using independent sample *t*-test

Bold values are remained significant even after bonferroni corrections

Sub-group analysis of the association between emotional function tests with different allergic disorders is shown in Table 3. The AR group scored less well than the non-atopic group on the DASS-21 test (depressed mood and anxiety behaviors), insomnia severity, as well as physical and mental health-related quality of life (*p* < 0.05). Additionally, the AD group obtained a higher score on the depression and stress scale compared to those without it (*p* < 0.05). Asthmatic patients also have significantly higher insomnia severity and lower physical health-related quality of life than non-asthmatic patients.

**Table 3:** Association between cognitive abilities and emotional function tests with different allergic disorders

	**Allergic rhino-conjunctivitis**			**Eczema**			**Asthma**		
	Yes 48(26.5%)	No 133(73.5%)	*p* value	Yes 27(14.9%)	No 154(85.1%)	*p* value	Yes 5(2.8%)	No 176(97.2%)	*p* value
*Dass-21*									
Depression	12.3 ± 9.6	9.2 ± 7.5	0.021	14.1 ± 9.7	10.2 ± 8.3	**0.035**	15.6 ± 9.3	10.8 ± 8.8	0.23
Anxiety	10.1 ± 6.9	7.4 ± 5.2	**0.005**	10.0 ± 8.0	8.6 ± 5.9	0.27	12.0 ± 8.2	8.8 ± 6.2	0.26
Stress	17.9 ± 10.1	16.7 ± 9.8	0.45	21.0 ± 11.2	16.6 ± 9.6	**0.037**	23.4 ± 6.5	17.2 ± 10.0	0.17
*Quality of life*									
Physical health	15.4 ± 2.6	16.2 ± 2.4	0.04	15.9 ± 2.4	15.8 ± 2.4	0.79	12.2 ± 4.4	15.8 ± 2.4	**0.001**
Mental health	15.8 ± 3.5	17.2 ± 3.8	0.011	15.9 ± 3.9	15.8 ± 2.4	0.41	14.2 ± 3.0	16.4 ± 3.7	0.18
SF-12 score	31.4 ± 5.0	33.5 ± 4.9	**0.006**	31.9 ± 5.5	32.4 ± 5.0	0.61	29.2 ± 3.0	32.3 ± 5.1	0.23
*Sleep pattern*									
Insomnia score (ISI)	7.4 ± 6.9	4.5 ± 6.3	**0.005**	6.0 ± 7.1	6.1 ± 6.6	0.96	13.6 ± 10.1	6.0 ± 6.7	**0.015**
Daytime sleepiness score (ESS)	6.8 ± 6.1	5.8 ± 5.7	0.20	6.4 ± 6.1	6.2 ± 5.9	0.85	9.4 ± 6.7	6.2 ± 5.9	0.23

Data presented as Mean ± SD

By using independent sample *t*-test

Bold values are remained significant even after bonferroni corrections

Binary regression analysis was performed to assess the relationship of having allergy and emotional complications. In logistic regression analyses adjusted for potential confounders, individuals that did not have allergy served as a reference group (Table 4). Those with at least one allergy disorders were more likely than healthy individuals to have anxiety behavior (OR = 1.86, 95% confidence interval [CI]:1.02–3.4), and insomnia symptoms (OR = 2.3; 95% CI 1.2–4.3).

**Table 4:** Logistic regression analysis of having any allergy as a predictor of anxiety behavior, low quality of life as well as insomnia

	Anxiety behavior	Low quality of life	Insomnia symptom
No-allergy	Ref	Ref	Ref
Having allergy	1.86(1.02–3.4)	1.75(0.88–3.48)	2.3(1.2–4.3)

Odds ratios with 95% confidence intervals (95% CI) obtained from binary logistic regression tests. Adjusted for age, BMI and energy intake

## Discussion

This is the first study that has investigated the association between cognitive abilities and emotional function and different allergic disorders in young women. In this cross-sectional study, we found that women who have any allergy have a greater degree of anxiety and insomnia, and have lower mental quality of life compared to those without it. Specifically, individuals with AR have higher depression, anxiety, insomnia and lower both mental and physical health score of quality of life; AD cases obtained a higher score on the depression and stress; and asthmatic patients also have significantly higher insomnia severity and lower physical health-related quality of life scale compared to those without it.

AR is not only a nasal problem but also leads to a wide spectrum of manifestations, some of which may lead to depression, anxiety, and insomnia problems related to reductions in mental and physical aspects of quality of life. AR is not only limited to the physical symptoms of the nose and eyes but also exacerbates disturbances in the well-being of the affected individuals.

Our findings are in accord with previous studies that have demonstrated that the depression risk in AR patients is higher than patients without AR [14, 39, 40]. It has been shown that the severity of depression was associated to disease activity and was even more profound when atmospheric pollen is high in individuals with seasonal AR [41]. AR patients most frequently suffer from sleep disturbances [42]. Among the several mechanisms influencing sleep quality/quantity, nasal congestion is an important feature. Resistance to air flux through the nasal airway causes a risk of sleep respiratory complications i.e. apnea or snoring [43].

Our results support previous findings that disturbed sleep and reduced health-related quality of life due to the physical and psychological impairments in AR patients [44, 45]. There is accumulating evidence indicating that individuals with AR have impaired psychological health and that these usually go undetected/untreated, and thus may causes mental distress [46–48]. AR symptoms such as ocular symptoms and nasal congestion can significantly disturb health-related quality of life and may make adults and children vulnerable to different comorbid conditions, which may further impair the quality of life [49].

In our population sample, 14.9% had eczema which was associated with greater levels of depression, and stress compared to those without it, and this is consistently supported in other studies [50, 51]. In a recent systematic review and meta-analysis, patients with eczema had a higher risk of depression. Increased duration and frequency of eruptions, as well as the increment in sick leave, were associated with higher psychological disorders [51].

Our results showed that asthmatic patients had a higher level of insomnia symptoms and lower physical health quality of health. The prevalence of insomnia among asthma patients varied from 10 to 70% in different population [52, 53]. Our results strengthen previous findings reported in asthmatic patients in which sleep disturbance was linked with lower disease-specific quality of life. Sleep problems are commonly related to an effect of nocturnal awakenings because of the nighttime asthma symptoms or the requirement for rescue inhaler medication [54, 55].

Other factors may also participate in the evolution of insomnia in asthmatic patients. Predisposing causes elevate the subject’s susceptibility to develop insomnia including personality characteristics i.e. anxiety or hyper-arousal, or genetic reasons such as female gender, aging, or family history [56]. Precipitating factors (stressful life events and health/psychological complications) can stimulate the onset of insomnia.

We have also shown that individuals who have at least one allergy suffer from higher anxiety and insomnia symptoms, and have lower mental health related quality of life. Multiple mechanisms have been suggested to explain allergies can affect mood. There may be somatic alterations such as discomfort induced by allergic inflammatory processes throughout the upper airway, which may influence well-being. This condition may causes to other possible mediators influencing mood, such the administration of medications (i.e. vasoconstrictors and antihistamines) or interfering with sleep induced by several factors such as airways obstruction [57, 58]. The release of cytokines as an inflammatory mediators possibly triggers mood worsening through direct action on the brain or via indirect other pathways such as interplays with the HPA axis and the IDO enzyme. It has been found that cytokines can promote anxiety and depression. Additionally, allergic symptoms have been related with the levels of cytokines released within some allergic responses [59]. Nevertheless, to the best of our knowledge, few studies evaluate the association of cognitive performances and symptom of allergic disorders; hence, this study provides worth information in this context. The results of the present study are consistent with those of previous research that has indicated that asthma, AR and AD does not alter cognitive abilities or practical IQ [60, 61].

This study has several limitations. It is a cross-sectional study, so it is not possible to conclude anything about the direction of the causation or the mechanism behind the relationship between allergy and depression. Future large longitudinal studies should be performed to support these findings and an investigation of the mechanisms attributed to this reported association. Notably, all of cognitive abilities and emotional function tests was measured only for the past 12 months, rather than lifetime.

## Conclusions

The results of present study showed that there was a high prevalence of psychological and psychiatric disorders such as anxiety, sleep problems as well as lower quality of life among allergic patients. Regarding the high prevalence of psychological problems in atopic patients, careful evaluation and treatment of these co-morbidities should be considered in patients visit.

## Data Availability

The datasets used and analyzed during the current study are available from the corresponding author on reasonable request.

## Abbreviations

AR: Allergic rhinitis
AD: Atopic dermatitis
IgE: Immunoglobulin E
CNS: Central nervous system
CAQ: Cognitive abilities questionnaire
DASS-21: Depression anxiety and stress scale
ESS: Epworth sleepiness scale
BMI: Body mass index
OR: Odds ratio
